# Psychosocial impact of maternal mortality on bereaved families and health care providers: what do we know?

**DOI:** 10.1007/s00404-026-08424-0

**Published:** 2026-04-16

**Authors:** Dana Alhaj, Selma Schmock, Lito-Laura Gerhold, Stephanie Wallwiener, Jarmila Zdanowicz, Claudia Hanson, Joachim Dudenhausen, Josefine Königbauer

**Affiliations:** 1 Department of Obstetrics, Charité-Universitätsmedizin Berlin, 10117 Berlin, Germany; 2 Department of Obstetrics, Fetal Medicine University Hospital Halle (Saale), Halle, Germany; 3 Department of Obstetrics and Gynecology, Inselspital, Bern University Hospital, University of Bern, Bern, Switzerland; 4 Department of Public Health Sciences, Karolinska Institutet, Stockholm, Sweden; 5 Department of Disease Control, London School of Hygiene and Tropical Medicine, London, UK; 6 GeMoRe digital registry German Obstetric Mortality Review, http://www.gemore-deutschland.eu

**Keywords:** Maternal mortality, Psychosocial impact, Bereaved families, Child health outcomes, Health care personnel, Midwives, Grief, Post-traumatic stress disorder, Coping strategies, Maternal death

## Abstract

**Introduction:**

Maternal mortality remains a critical global health issue with profound psychosocial consequences that extend beyond the deceased woman to her family and the healthcare professionals (HCP) involved in her care. While substantial progress has been made in reducing maternal mortality worldwide, its social and psychological sequelae remain insufficiently studied, particularly in high-income countries.

**Methods:**

This narrative review is based on a structured literature search conducted in PubMed and Google Scholar for studies published between 2000 and 2025. Study selection was guided by predefined inclusion criteria, and relevant articles were identified through keyword searches and snowballing. Data were extracted and analyzed using a narrative thematic approach focusing on psychosocial outcomes in families and HCP.

**Results:**

The available evidence, predominantly derived from qualitative and mixed-methods studies in low- and middle-income countries—especially sub-Saharan Africa—demonstrates consistent patterns of psychological distress, social disruption, and long-term adverse outcomes among affected families. Children are particularly vulnerable to educational, emotional, and economic disadvantages following maternal death. For HCP, particularly midwives, maternal mortality is associated with significant emotional burden, including guilt, grief, and professional self-doubt, as well as social and occupational consequences.

**Discussion:**

Despite the global relevance of maternal mortality, there is a marked lack of data from high-income settings. Existing findings suggest that both families and HCP experience substantial and enduring psychosocial impacts, yet structured institutional support systems are often lacking.

**Conclusion:**

Maternal mortality has far-reaching psychosocial consequences for families and HCP alike. The findings highlight the urgent need for targeted support interventions, structured training, and further research, particularly in high-income countries, to better understand and mitigate these effects.

## Introduction

Maternal mortality is defined by the World Health Organization (WHO) as the death of a woman during pregnancy or within 42 days after delivery, from any cause related to or aggravated by the pregnancy or its management, but not from accidental or incidental causes [1]. Despite sustained global efforts to improve maternal health, maternal mortality remains a major public health challenge. Since 2000, the global number of maternal deaths has declined by approximately 40%; however, an estimated 260,000 women still died worldwide from maternal causes in 2023, underscoring the persistent burden of preventable maternal deaths [2]. Striking regional inequalities continue to characterize maternal mortality, with nearly 70% of maternal deaths occurring in sub-Saharan Africa [2, 3]. Maternal mortality ratios (MMR) in low-income countries frequently exceed 346 per 100,000 live births, in sharp contrast to high-income countries, where ratios are typically below 10 per 100,000 live births [2]. These disparities reflect profound differences in access to quality obstetric care, health system capacity, and broader social determinants of health.

Research on maternal mortality has traditionally focused on its epidemiology, medical causes, and prevention strategies. Globally, obstetric hemorrhage, hypertensive disorders of pregnancy, and indirect causes such as pre-existing medical conditions remain the leading contributors to maternal death although their relative importance varies considerably across regions [4]. While this research has been instrumental in informing clinical guidelines and public health interventions, it has largely conceptualized maternal mortality as a biomedical outcome. In contrast, the psychosocial consequences of maternal death for surviving family members, children, and health care professionals (HCP) have received comparatively limited attention. Maternal death represents not only the loss of a woman during a critical life stage but also a devastating social and psychological event, with potential long-term effects on family structures, child health and development, and the well-being of the health care workforce. Understanding these broader consequences is essential for developing comprehensive maternal health strategies that extend beyond mortality reduction to include psychosocial support, resilience, and sustainability of health care systems.

## Methods

### Literature search

We conducted a structured literature search to identify relevant publications on the psychosocial impact of maternal mortality. The databases PubMed and Google Scholar were searched for articles published between 2000 and 2025. The search strategy combined keywords related to the psychosocial impact on the family of the deceased woman and the HCP involved in her care, including terms, such as “maternal mortality”, “maternal death”, “psychosocial impact”, “family”, “children”, “health care providers”, and “midwives”. Many of the articles were found on Google Scholar, using the terms “impact of maternal death on midwives” and “impact of maternal death on families”. The other articles used were gathered later through snowballing, due to limited results from the primary search. We only included articles explicitly investigating the impact of maternal mortality; articles studying severe obstetric events under a broad definition were excluded. Only articles published in English and/or German were considered. The search was performed during the period 30 August 2025–30 September 2025.

### Study selection

Articles were selected based on their relevance to the scope of this review. Original research articles, as well as narrative and systematic reviews, were included. Many of the included articles were based on qualitative or mixed-methods studies. The search aimed to find studies focusing on the psychosocial effects of maternal mortality. The selection was based on title and abstract screening, followed by full-text assessment where appropriate. To ensure completeness, reference lists of selected articles were manually screened for additional relevant publications. Primary search and study selection were conducted by one person. Given the narrative review design, no other tools were implemented and no formal risk-of-bias assessment was performed.

### Data analysis

Data from the identified relevant studies were extracted using a structured approach, including study setting, population, study design, outcomes assessed, and key findings relevant to the review objectives. Given the heterogeneity of study designs and outcome measures, a quantitative synthesis was not appropriate. Instead, findings were analyzed using a narrative thematic approach. Reported results were compared across studies and grouped into recurring thematic domains related to psychological impact, social and economic consequences, health effects, and coping mechanisms of affected families and HCP, respectively.

## Results

The primary search for the impact on medical personnel yielded seven articles through title screening, and one of them was excluded after abstract screening. Six additional articles were found through reference list screening of the initially included studies; four of those were included after abstract screening.

**Fig. 1:**
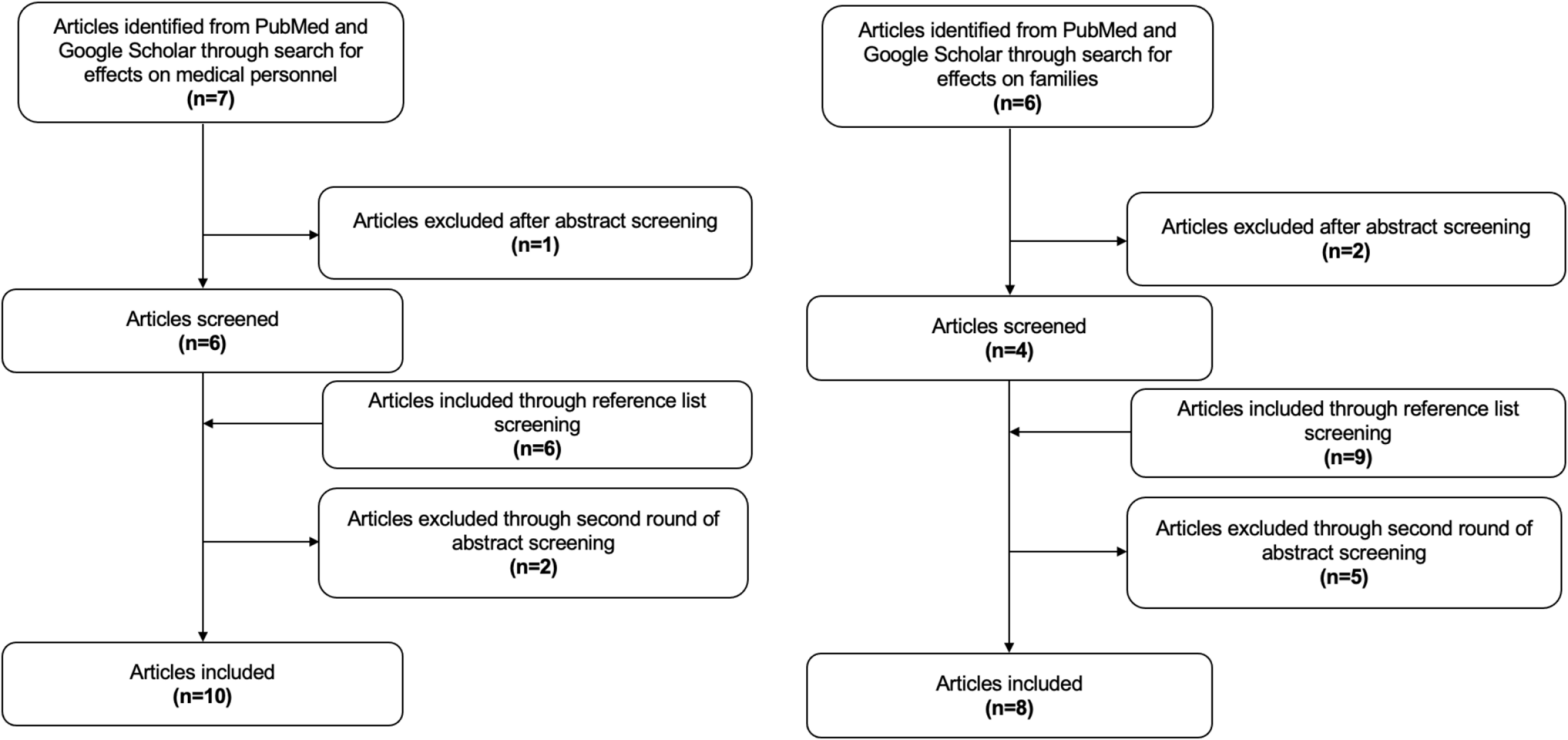
Flowcharts detailing the literature search for the effects of maternal death on medical personnel and affected families, respectively. A total of 18 articles were included in the review

With the search for the impact on the family, six studies were found through title screening, and two of them were excluded after abstract screening. We found nine additional studies by screening the references of these articles and included four of these after abstract screening. In conclusion, a total of 18 articles were included in the final narrative synthesis based on relevance to the review objectives. Figure 1 shows an overview of the respective literature search.

Most studies examining the effects on health care providers focused primarily on midwives, with comparatively few including other professional groups. 16 of the studies were conducted in sub-Saharan Africa, one in China, while only one originated from the United Kingdom. Methodologically, the literature consisted predominantly of qualitative and mixed-methods studies. The qualitative studies were predominantly using one-on-one interviews or group discussions, while the mixed-methods studies used questionnaires to gather quantitative information, for example to discover depression symptoms. The results are presented thematically in the following sections based on these findings.

### Psychosocial impact on families

#### Psychological consequences

In one of the studies, bereaved families reported profound grief, emotional numbness, and persistent sadness following maternal death. The mixed-methods research from Ghana demonstrated that more than half of family members exhibited mild depressive symptoms, as assessed by standardized instruments, such as the Patient Health Questionnaire-9 (PHQ-9) and the Inventory of Complicated Grief [5]. Fathers were identified as a particularly vulnerable group; a prospective cohort study from rural China found that over 50% of widowed fathers were at increased risk of post-traumatic stress disorder (PTSD) one year after the maternal death [6].

Substance use as a coping strategy was reported but appeared relatively uncommon. In Ghanaian cohorts, alcohol misuse affected fewer than 10% of respondents, and illicit drug use was rare [5]. Nonetheless, the psychological burden of loss was consistently described as enduring and compounded by ongoing caregiving responsibilities and economic stress [5, 7–11].

#### Social and economic consequences

Maternal death frequently leads to major disruptions in household structure. Studies from Malawi, Ethiopia, Kenya, and Tanzania describe a redistribution of domestic responsibilities, often affecting daughters disproportionately due to prevailing gender norms [7–10]. Children are frequently required to assume caregiving roles or contribute to household labor, leading to school absenteeism, delayed educational progress, or complete school dropout [7–10].

Economic hardship is a recurrent theme. The loss of maternal income, combined with high costs for medical care and burial, places families at substantial financial risk [5, 11]. In extended family arrangements, the redistribution of children can strain already limited household resources, reducing per-child expenditure on education and health [10]. Some families reported migration as a coping strategy in pursuit of better economic opportunities [8].

#### Health consequences

The health implications for children following maternal death are severe. Infant and child mortality rates are higher among children whose mothers died from maternal causes [8, 12]. Studies from Ethiopia and Haiti indicate markedly reduced survival beyond infancy, with undernutrition being a key contributing factor [8, 12]. Zhou et al. reported a fourfold increase in the risk of undernutrition among affected children compared with peers whose mothers survived [6]. Inadequate access to breast milk or appropriate infant formula further exacerbates these risks, particularly in low-resource settings [5, 8, 10].

#### Coping mechanisms

**Fig. 2:**
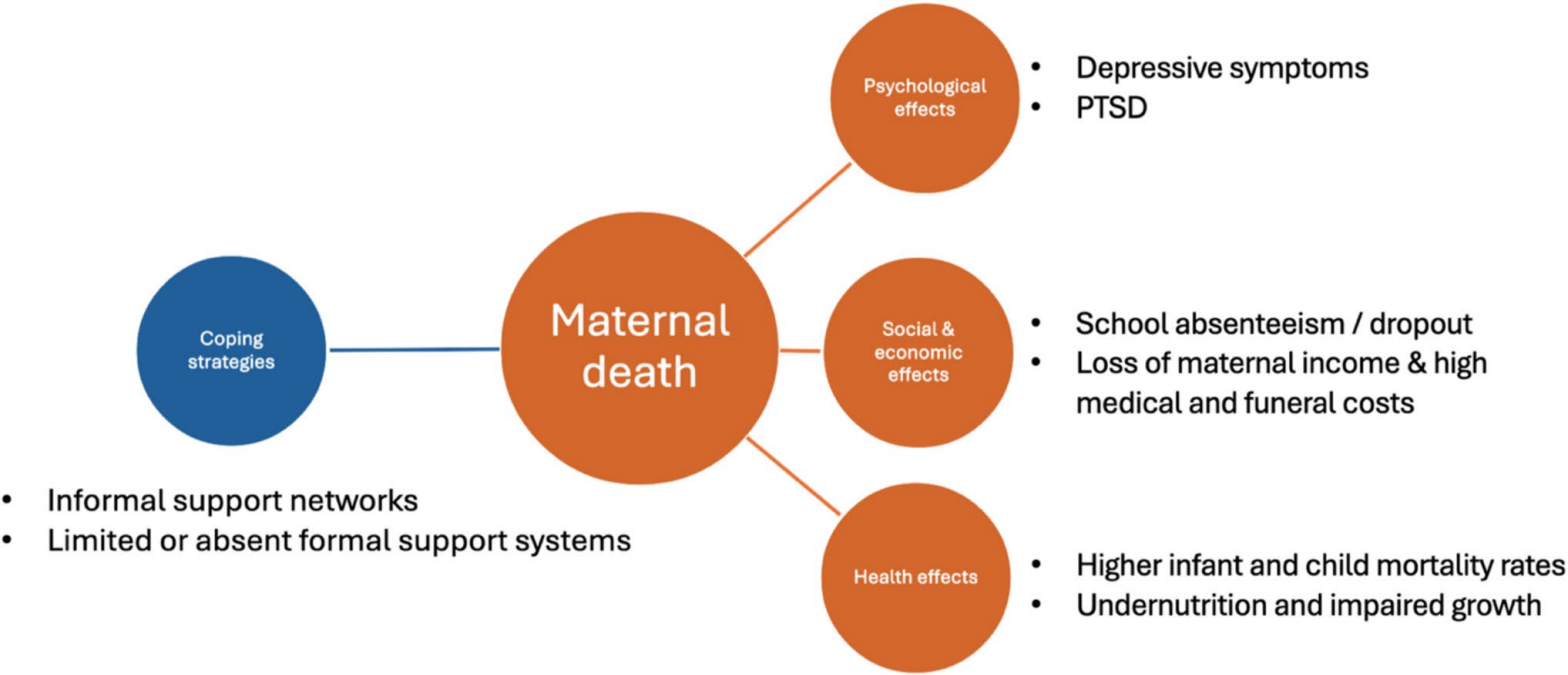
Effects of maternal death on bereaved families and coping strategies, including psychological, social and occupational, as well as health effects [5–12]

Families primarily rely on informal support networks, including extended family members, neighbors, religious communities, and friends including financial or emotional assistance [5, 8, 9]. Some participants stated that the support after the death had diminished over time [5, 9]. Across studies, there is a notable absence of structured, institutional support systems for bereaved families, highlighting a significant gap in post-maternal death care [8]. Figure 2 summarizes the effects of maternal death on families and possible coping strategies.

### Psychosocial impact on health care providers

#### Psychological effects

Health care providers, particularly midwives, experience maternal death as a traumatic professional event. Qualitative studies from Ghana, Uganda, Namibia, Lesotho, and the United Kingdom consistently describe intense feelings of guilt, grief, fear, and self-blame or shame [13–20]. High levels of death-related anxiety were reported, with some studies documenting symptoms consistent with depression and PTSD, including flashbacks, nightmares, panic attacks, and insomnia [14, 15, 18].

#### Social and occupational consequences

Fear of legal consequences, professional blame, and negative reactions from patients’ families further compound psychological distress [16, 20]. These fears may persist long after the event and influence future clinical decision-making.

Maternal death can thus negatively affect providers’ social functioning and professional identity. Some midwives reported social withdrawal, reduced engagement with family and friends, and diminished self-care [21]. A significant proportion questioned their career choice, with up to one quarter considering leaving the profession following such events [20].

#### Health consequences

Psychological stress is frequently accompanied by physical symptoms, including sleep disturbances, appetite loss, weight changes, and chronic fatigue or insomnia [17, 21]. These symptoms can impair work performance and contribute to burnout and reduced quality of care.

#### Coping strategies and institutional support

Collegial support emerged as the most frequently cited coping mechanism among health care providers [13, 14, 20, 22]. Informal discussions with colleagues, family support, and spirituality were commonly described as helpful [20, 22]. However, maladaptive coping strategies, including alcohol use, were also reported in some contexts [17].

**Fig. 3:**
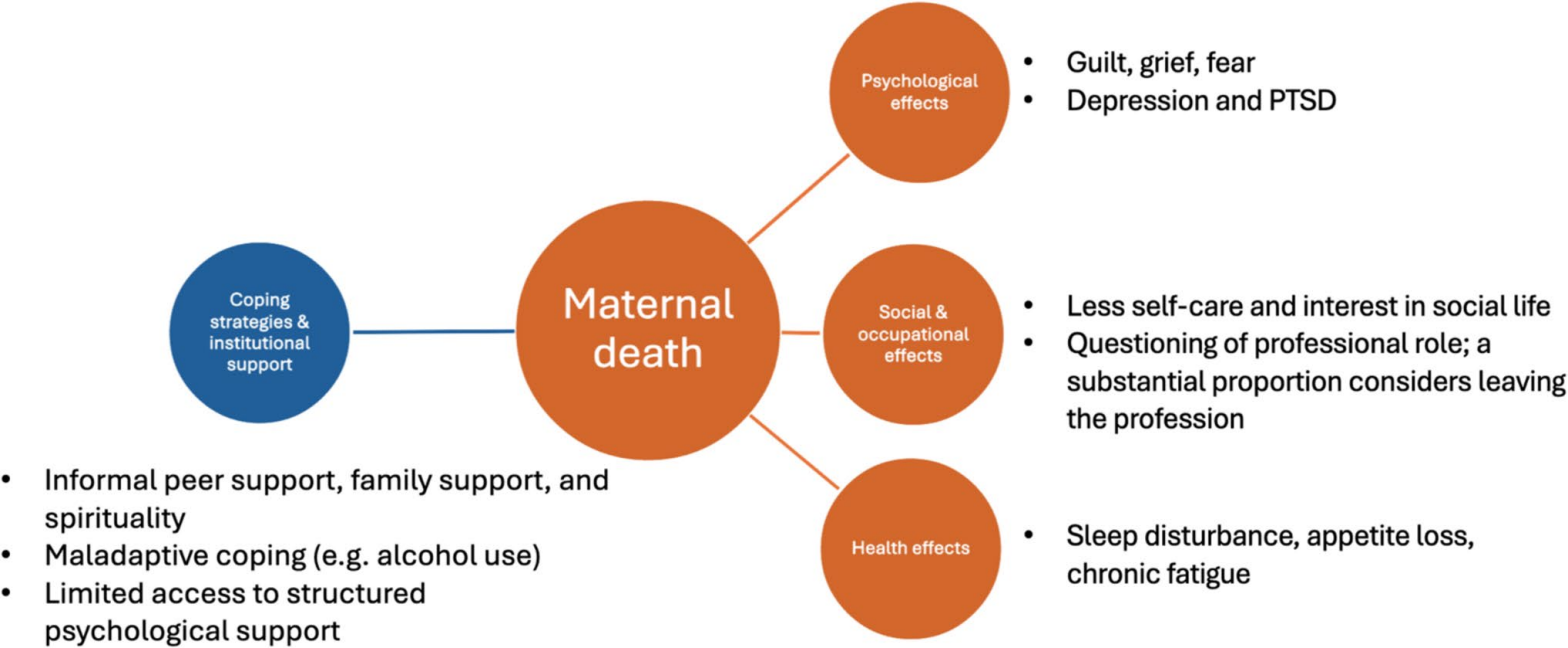
Effects of maternal death on health care providers and coping strategies, including psychological, social and occupational, as well as health effects [13–22]

Institutional responses varied widely. In high-income settings, such as the United Kingdom and a study from Ghana, structured debriefings and review meetings following maternal death were described as potentially beneficial, although they sometimes involved finger-pointing [13, 14]. Across settings, providers consistently expressed a need for formal psychological support and targeted training addressing both clinical management and emotional coping. [13, 14] Evidence suggests that coping-focused training substantially improves perceived preparedness and resilience [20]. Figure 3 shows an overview of the consequences of maternal death on health care providers and observed coping strategies.

## Discussion

This narrative review underlines that maternal mortality has profound and far-reaching psychosocial consequences for both affected families and health care providers. Despite substantial heterogeneity in study designs, populations, and health system contexts, the findings across studies are generally consistent.

Maternal death is repeatedly associated with long-term psychological distress, social disruption, economic insecurity, and adverse health and developmental outcomes for surviving children. Particularly striking is the increased rate of infant and child mortality following maternal death; a study from Haiti reported 55% increased odds of experiencing the death of a child under 12 years of age [12]. Several studies report malnutrition and wasting of surviving infants as a result of early termination of breastfeeding but also of increased economic burden [6, 8, 12]. Molla et al. [8] draw on the perception of mothers as “primary nurtures of and advocates for their children” in their discussion of the grave effects of maternal death on all aspects of the surviving family’s life; they conclude by emphasizing the importance of social welfare programs to support bereaved families and of improved obstetric care to avert the “human tragedy” of maternal mortality.

Beyond its impact on families, maternal death also emerges as a significant occupational trauma for health care providers, with documented implications for mental well-being, professional identity, job satisfaction, and staff shortages. Contributing to these negative effects on HCP are feelings of guilt as well as fear of disciplinary or legal consequences [16, 20]. A systematic review of 59 studies on maternal death surveillance and response [23] emphasizes the role of a blame-free learning environment in effecting positive outcomes of surveillance; fear of negative consequences following maternal death was shown to lead to negative outcomes including under-reporting, falsification of information, and in some cases refusal to accept referrals in order to avoid responsibility [23]. Evidence from studies on effects of maternal death on families as well as on HCP underscores the importance of structured, institutional support systems.

The existing body of literature is predominantly qualitative in nature and is heavily concentrated in sub-Saharan Africa. As a result, the transferability of findings to other settings, particularly high-income countries, is limited due to substantial differences in cultural norms, social contexts, professional roles, and health system structures. Furthermore, there is a notable lack of comparative studies that include physicians alongside midwives, restricting conclusions about profession-specific or interdisciplinary effects. Many studies are characterized by small sample sizes, which may reduce the robustness of findings, and the use of standardized and validated outcome measures is inconsistent across studies. Collectively, these limitations constrain the generalizability and comparability of the available evidence and highlight the need for more methodologically rigorous and geographically diverse research.

Nevertheless, the available evidence suggests that even in contexts where maternal death is relatively rare, its psychosocial impact on health care providers may be substantial and enduring. The scarcity of data from high-income settings therefore represents a critical gap in the literature and underscores the need for context-specific research to inform targeted prevention and support strategies.

## Conclusions

Maternal mortality is a relatively rare but devastating event in high-income countries and a persistent public health challenge in low-resource settings. Beyond its immediate medical implications, maternal death exerts deep and lasting psychosocial effects on families and health care providers. The findings of this review highlight the urgent need for structured support systems, dedicated training programs, and further research, particularly in high-income settings, to mitigate the long-term consequences of maternal mortality and to support both bereaved families and the professionals who care for them. To address this research gap, we are planning a multicenter study in Europe. We will use semi-structured interviews and detailed questionnaires to explore the experiences of medical personnel of different professions with maternal mortality and other severe obstetric events with the aim of introducing targeted support strategies to improve overall obstetric care.

## Data Availability

The data supporting the conclusions of this article are included or cited in this published article.

## Abbreviations

HCP: Health care professionals
WHO: World Health Organization
MMR: Maternal mortality rate
PHQ-9: Patient Health Questionnaire-9
PTSD: Post-traumatic stress disorder

## References

[1] World Health Organization (2012) WHO Application of ICD-10 to Deaths during Pregnancy Childbirth and Puerperium. World Health Organization, ICD-MM. Geneva

[2] Maternal mortality. https://www.who.int/news-room/fact-sheets/detail/maternal-mortality Accessed 25 October 2025.

[3] Roser M, Ritchie H (2013) Maternal Mortality. Our World Data. https://ourworldindata.org/maternal-mortality, Accessed 24 November 2025.

[4] Cresswell JA, Alexander M, Chong MYC, Link HM, Pejchinovska M, Gazeley U et al (2025) Global and regional causes of maternal deaths 2009–20: a WHO systematic analysis. Lancet Glob Health 13(4):e626–e634 40064189 10.1016/S2214-109X(24)00560-6 PMC11946934

[5] Lawrence ER, Appiah-Kubi A, Lawrence HR, Lui MY, Owusu-Antwi R, Konney T et al (2022) “There is no joy in the family anymore”: a mixed-methods study on the experience and impact of maternal mortality on families in Ghana. BMC Pregnancy Childbirth 22:683 36064376 10.1186/s12884-022-05006-1 PMC9443015

[6] Zhou H, Zhang L, Ye F, Jun WH, Huntington D, Huang Y et al (2016) The effect of maternal death on the health of the husband and children in a rural area of china: a prospective cohort study. PLoS ONE 11(6):e0157122 27280717 10.1371/journal.pone.0157122 PMC4900542

[7] Bazile J, Rigodon J, Berman L, Boulanger VM, Maistrellis E, Kausiwa P et al (2015) Intergenerational impacts of maternal mortality: qualitative findings from rural Malawi. Reprod Health 12(S1):S1 26000733 10.1186/1742-4755-12-S1-S1 PMC4423580

[8] Molla M, Mitiku I, Worku A, Yamin AE (2015) Impacts of maternal mortality on living children and families: a qualitative study from Butajira, Ethiopia. Reprod Health 12(S1):S6 26001276 10.1186/1742-4755-12-S1-S6 PMC4423766

[9] Pande RP, Ogwang S, Karuga R, Rajan R, Kes A, Odhiambo FO et al (2015) Continuing with “…a heavy heart”—consequences of maternal death in rural Kenya. Reprod Health 12(S1):S2 26000827 10.1186/1742-4755-12-S1-S2 PMC4423749

[10] Yamin AE, Boulanger VM, Falb KL, Shuma J, Leaning J (2013) Costs of inaction on maternal mortality: qualitative evidence of the impacts of maternal deaths on living children in Tanzania. PLoS ONE 8(8):e71674 23990971 10.1371/journal.pone.0071674 PMC3747181

[11] Kes A, Ogwang S, Pande RP, Douglas Z, Karuga R, Odhiambo FO et al (2015) The economic burden of maternal mortality on households: evidence from three sub-counties in rural western Kenya. Reprod Health 12(Suppl 1):S3 26000953 10.1186/1742-4755-12-S1-S3 PMC4423575

[12] Anderson FWJ, Morton SU, Naik S, Gebrian B (2007) Maternal mortality and the consequences on infant and child survival in rural Haiti. Matern Child Health J 11(4):395–401 17265193 10.1007/s10995-006-0173-0

[13] Cauldwell M, Chappell LC, Murtagh G, Bewley S (2015) Learning about maternal death and grief in the profession: a pilot qualitative study. Acta Obstet Gynecol Scand 94(12):1346–1353 26332761 10.1111/aogs.12760

[14] Stabnick A, Yeboah M, Arthur-Komeh J, Ankobea F, Moyer CA, Lawrence ER (2022) “Once you get one maternal death, it’s like the whole world is dropping on you”: experiences of managing maternal mortality amongst obstetric care providers in Ghana. BMC Pregnancy Childbirth 22(1):206 35287601 10.1186/s12884-022-04535-z PMC8919901

[15] Muliira RS, Bezuidenhout MC (2015) Occupational exposure to maternal death: psychological outcomes and coping methods used by midwives working in rural areas. Midwifery 31(1):184–190 25217107 10.1016/j.midw.2014.08.005

[16] Dartey AF (2017) Fears associated with maternal death: selected midwives’ lived experiences in the Ashanti Region of Ghana. Numid Horizon Int J Nurs Midwifery 1(1):79–86

[17] Mohale L, Nyangu I (2023) Psychological experiences of midwives regarding maternal deaths at two selected public hospitals in Lesotho. New Voices Psychol. https://unisapressjournals.co.za/index.php/NV/article/view/15144. Accessed 29 September 2025.

[18] Endjala T, Amukugo HJ, Ngitanwa EM (2021) Post-traumatic stress disorder among midwives after exposure to maternal death and stillbirth in Khomas region of Namibia. Int J Healthc 7(2):7

[19] Dartey AF, Phetlhu DR, Phuma-Ngaiyaye E (2019) Coping with maternal deaths: the experiences of midwives. Ethiop J Health Sci 29(4):495–502 31447523 10.4314/ejhs.v29i4.11 PMC6689706

[20] Lawrence ER, Stabnick A, Arthur‐Komeh J, Moyer CA, Yeboah M (2021) Preparedness to deal with maternal mortality among obstetric providers at an urban tertiary hospital in Ghana. Int J Gynaecol Obstet 154(2):358–365 33314104 10.1002/ijgo.13537

[21] Dartey AF, Phuma-Ngaiyaye E (2020) Physical effects of maternal deaths on midwives’ health: a qualitative approach. J Pregnancy 2020(1):2606798 32308995 10.1155/2020/2606798 PMC7152977

[22] Dartey AF, Phetlhu DR, Phuma-Ngaiyaye E (2019) Reducing the effects of maternal death: midwives’. Int J Health Sci Res 9(2):128–138

[23] Willcox M, Okello I, Maidwell-Smith A, Tura A, van den Akker T, Knight M (2023) Maternal and perinatal death surveillance and response: a systematic review of qualitative studies. Bull World Health Organ. 10.2471/blt.22.288703 36593778 10.2471/BLT.22.288703 PMC9795385

